# Empirical and deterministic accuracies of across-population genomic prediction

**DOI:** 10.1186/s12711-014-0086-0

**Published:** 2015-02-06

**Authors:** Yvonne CJ Wientjes, Roel F Veerkamp, Piter Bijma, Henk Bovenhuis, Chris Schrooten, Mario PL Calus

**Affiliations:** Animal Breeding and Genomics Centre, Wageningen UR Livestock Research, 6700 AH Wageningen, The Netherlands; Animal Breeding and Genomics Centre, Wageningen University, 6700 AH Wageningen, The Netherlands; CRV BV, 6800 AL Arnhem, The Netherlands

## Abstract

**Background:**

Differences in linkage disequilibrium and in allele substitution effects of QTL (quantitative trait loci) may hinder genomic prediction across populations. Our objective was to develop a deterministic formula to estimate the accuracy of across-population genomic prediction, for which reference individuals and selection candidates are from different populations, and to investigate the impact of differences in allele substitution effects across populations and of the number of QTL underlying a trait on the accuracy.

**Methods:**

A deterministic formula to estimate the accuracy of across-population genomic prediction was derived based on selection index theory. Moreover, accuracies were deterministically predicted using a formula based on population parameters and empirically calculated using simulated phenotypes and a GBLUP (genomic best linear unbiased prediction) model. Phenotypes of 1033 Holstein-Friesian, 105 Groninger White Headed and 147 Meuse-Rhine-Yssel cows were simulated by sampling 3000, 300, 30 or 3 QTL from the available high-density SNP (single nucleotide polymorphism) information of three chromosomes, assuming a correlation of 1.0, 0.8, 0.6, 0.4, or 0.2 between allele substitution effects across breeds. The simulated heritability was set to 0.95 to resemble the heritability of deregressed proofs of bulls.

**Results:**

Accuracies estimated with the deterministic formula based on selection index theory were similar to empirical accuracies for all scenarios, while accuracies predicted with the formula based on population parameters overestimated empirical accuracies by ~25 to 30%. When the between-breed genetic correlation differed from 1, i.e. allele substitution effects differed across breeds, empirical and deterministic accuracies decreased in proportion to the genetic correlation. Using a multi-trait model, it was possible to accurately estimate the genetic correlation between the breeds based on phenotypes and high-density genotypes. The number of QTL underlying the simulated trait did not affect the accuracy.

**Conclusions:**

The deterministic formula based on selection index theory estimated the accuracy of across-population genomic predictions well. The deterministic formula using population parameters overestimated the across-population genomic accuracy, but may still be useful because of its simplicity. Both formulas could accommodate for genetic correlations between populations lower than 1. The number of QTL underlying a trait did not affect the accuracy of across-population genomic prediction using a GBLUP method.

## Background

For genomic prediction, a reference population that consists of individuals with phenotypes and marker genotypes is used to estimate marker effects and to predict breeding values for another group of genotyped individuals, called selection candidates. The accuracy of predicting breeding values for selection candidates within one population is influenced by the level of linkage disequilibrium (LD) between markers, i.e. single-nucleotide polymorphisms (SNPs) and quantitative trait loci (QTL) that influence the trait, and by the level of family relationships [1-4]. Across populations, there are differences in LD, allele frequencies [4-6], and allele substitution effects of QTL [7,8], and close family relationships between individuals of different populations are absent. Therefore, the potential accuracy of predicting breeding values when the predicted population is not included in the reference population is likely to be limited. Indeed, in dairy cattle breeding, several empirical studies showed that the potential of using information across breeds was limited, e.g. [9-11]. The concept of combining individuals of different breeds in cattle is essentially similar to combining individuals from different lines in other animal and plant species, e.g. [4,12,13] or from different subpopulations in humans, e.g. [3,6] because close family relationships are absent and the extent of LD is limited across breeds, lines, and subpopulations.

A higher marker density may increase the consistency in LD phase across populations, since at short distances (5 to 30 kb) LD phases are conserved across populations [5]. However, several empirical studies showed that an increase in marker density resulted only in a small increase in accuracy using multiple populations in the reference population [14,15]. This small effect of marker density on accuracy indicates that other factors are also important, such as differences in segregating QTL or in the effect of QTL across populations due to differences in genetic background between populations [7,8]. *DGAT1* (*diacylglycerol O-acyltransferase 1*) is one example of a gene with different effects across populations in dairy cattle. Allele substitution effects of a QTL in the *DGAT1* locus on milk yield and fat yield have been found to be on average 0.8 and 0.5 times, respectively, as large in Jersey than in Holstein-Friesian populations in New Zealand [7] and 0.7 and 1.2 times, respectively, as large in Fleckvieh than in Holstein-Friesian populations in Germany [8]. Since the SNP that was analysed is considered to be the causal polymorphism, which rules out incomplete LD, these results demonstrate that large differences in allele substitution effects can exist across populations.

Another factor that may affect accuracy of genomic prediction across populations is the number of QTL underlying the trait. For genomic prediction based on one population, accuracy is shown to be independent of the number of QTL underlying the trait when a genomic best linear unbiased prediction method (GBLUP) is used [16,17], at least in situations for which there are no QTL that explain an extremely large part of the genetic variance. However, those studies only looked at the effect of the number of QTL on accuracy of genomic prediction within one population and not across populations.

For genomic prediction within one population, different deterministic formulas have been proposed to calculate the accuracy [1,2]. The formula of Daetwyler *et al.* [1] uses population and trait parameters, i.e. size of the reference population, heritability and number of effective chromosome segments. If the number of effective chromosome segments is calculated from the variation of genomic relationships around their expectations based on pedigree information, the formula of Daetwyler *et al.* [1] can also be applied for populations with a complex family structure [18]. The formula of VanRaden [2] can be derived both from selection index theory and prediction error variance of the mixed model equation and it estimates the accuracy using the relationships within the reference population and between selection candidates and the reference population. Hayes *et al*. [9] showed that applying the formula based on prediction error variance in multi-population situations without rescaling the genomic relationships across populations resulted in overestimation of the accuracy. This indicates that formulas for estimating the accuracy of genomic prediction using multiple populations need further investigation to define the best way to calculate genomic relationships across populations.

The first objective of this study was to develop a deterministic formula to estimate the accuracy of across-population genomic prediction. The second objective was to investigate the effect of differences in allele substitution effects of QTL across populations on accuracy of across-population genomic prediction. The last objective was to investigate the effect of the number of QTL underlying a trait on accuracy of across-population genomic prediction. Two deterministic formulas were evaluated and empirical accuracies were calculated using simulated phenotypes based on real genotypes from three cattle breeds representing different populations. Phenotypes were simulated using different correlations between allele substitution effects across breeds and different numbers of QTL underlying the trait. The reason for simulating the phenotypes of the individuals was to be able to investigate the actual effects of differences in allele substitution effects of QTL across populations and of the number of QTL by changing one factor at a time without changing the other factors, which would not be possible with real data.

## Methods

### Across-population genomic prediction

For genomic prediction based on one population, breeding values are predicted for individuals using a reference population of individuals from the same population. In most genomic prediction models, the QTL effects that underlie the traits of interest are assumed to be additive, e.g. [19]. For across-population genomic prediction, breeding values are predicted for individuals using a reference population of individuals from one or more different populations. Due to differences in allele frequencies across populations, the presence of non-additive effects can result in differences in allele substitution effects of QTL [20]. Therefore, the models used for across-population genomic prediction should include non-additive effects or allow for differences in allele substitution effects across populations. Since it is difficult to accurately estimate non-additive effects, e.g. [21,22], assuming additive gene action and, at the same time, allowing for differences in allele substitution effects may be a good first step and is the focus of this study. The correlation between allele substitution effects across populations can be considered as the genetic correlation between the populations [20,23].

Based on the assumption of additive QTL effects and using selection index theory, the breeding value of individual *i* of population *A* can be predicted using reference population *B* as:

1 $$ {\widehat{a}}_{A_i}=\mathbf{b}{\hbox{'}}_{AB}{\mathbf{y}}_B=Cov\left({a}_{A_i},{\mathbf{y}}_B\right){\left[Var\left({\mathbf{y}}_B\right)\right]}^{-1}{\mathbf{y}}_B, $$

where ${\widehat{a}}_{A_i}$ is the predicted breeding value of individual *i* of population *A*, **b**_*AB*_ is a *n*_*B*_ x1 vector with partial regression coefficients of breeding values of population *A* on phenotypes of population *B*, **y**_*B*_ is a *n*_*B*_ x1 vector with phenotypes corrected for fixed effects of individuals from population *B*, ${a}_{A_i}$ is the true breeding value of individual *i* of population *A*, and *n*_*B*_ is the number of individuals in reference population *B*.

The covariance between the true breeding value (TBV) of individual *i* of population *A* and the phenotypes of individuals from population *B* is:

2 $$ \begin{array}{l}Cov\left({a}_{A_i},{\mathbf{y}}_B\right)=Cov\left({a}_{A_i},{\mathbf{a}}_B+{\mathbf{e}}_B\right)\\ {}=Cov\left({a}_{A_i},{\mathbf{a}}_B\right)+Cov\left({a}_{A_i},{\mathbf{e}}_B\right),\end{array} $$

where **a**_*B*_ is a *n*_*B*_ x1 vector with TBV of individuals from population *B* and **e**_*B*_ is a *n*_*B*_ x1 vector with environmental effects of individuals from population *B*. In an additive model *Cov* (**a**, **e**) = 0, Equation 2 reduces to:

3 $$ Cov\left({a}_{A_i},{\mathbf{y}}_B\right)=Cov\left({a}_{A_i},{\mathbf{a}}_B\right)={r}_{G_{AB}}{\upsigma}_{a_A}{\upsigma}_{a_B}\mathbf{g}{\hbox{'}}_{A_i,B}, $$

where ${r}_{G_{AB}}$ is the genetic correlation between population *A* and population *B*, ${\upsigma}_{a_A}$ and ${\upsigma}_{a_B}$ are the genetic standard deviations in populations *A* and *B*, respectively, ${\mathbf{g}}_{A_i,B}$ is a *n*_*B*_ x1 vector with genomic relationships between individual *i* of population *A* and reference individuals of population *B*.

Under the assumption that SNPs are representative of QTL, i.e. that characteristics such as allele frequency are the same for SNPs and QTL, resulting in usable LD between SNPs and QTL, a genomic relationship matrix based on SNPs can be used to represent the relationships between breeding values of the individuals. To calculate the genomic relationships, covariances between the individuals of both populations need to be calculated. The mathematical definition of a covariance, $Cov\left(x,y\right)=E\left[\left(x-\overline{x}\right)\left(y-\overline{y}\right)\right]$, indicates that both components are corrected for their own mean. For the genomic relationships, this can be achieved by correcting the SNP genotypes of the individuals using the allele frequencies of their own population. Thus, the genotype of individual *i* from population *j* at locus *k*, *g*_*ijk*_, is standardized as ${x}_{ijk}=\frac{g_{ijk}-2{p}_{jk}}{\sqrt{2{p}_{jk}\left(1-{p}_{jk}\right)}}$, where *p*_*jk*_ is the allele frequency of population *j* at locus *k*, and the standardized genotypes are used to calculate the genomic relationship matrices using the method of Yang *et al*. [24], which will be described later.

Hence, Equation 1 can be written as:

4 $$ {\widehat{a}}_{A_i}={r}_{G_{AB}}{\upsigma}_{a_A}{\upsigma}_{a_B}\mathbf{g}{\hbox{'}}_{A_i,B}{\left[Var\left({\mathbf{y}}_B\right)\right]}^{-1}{\mathbf{y}}_B. $$

This expression for the estimated breeding value (EBV) will subsequently be used in the next section to derive the accuracy.

### Deterministic accuracy of across-population genomic prediction based on selection index theory

The general formula to calculate the accuracy of prediction of a breeding value is [20]:

5 $$ {r}_{A_i}=\frac{Cov\left({\widehat{a}}_{A_i},{a}_{A_i}\right)}{\sqrt{Var\left({a}_{A_i}\right)Var\left({\widehat{a}}_{A_i}\right)}} $$

In single-population situations, it is well known that $Cov\left({\widehat{a}}_{A_i},{a}_{A_i}\right)=Var\left({\widehat{a}}_{A_i}\right)$ [20]. This is also correct for across-population genomic prediction, as shown in the Appendix. Therefore, the expression for the accuracy of across-population genomic prediction reduces to:

6 $$ {r}_{A_i}=\sqrt{\frac{Cov\left({\widehat{a}}_{A_i},{a}_{A_i}\right)}{Var\left({a_A}_i\right)}}. $$

The covariance between the predicted and TBV of individual *i* of population *A* can be calculated as (see Appendix):

7 $$ \begin{array}{l}Cov\left({\widehat{a}}_{A_i},{a}_{A_i}\right)\\ {}={r}_{G_{AB}}^2{\upsigma}_{a_A}^2{\upsigma}_{a_B}^2\mathbf{g}{\hbox{'}}_{A_i,B}{\left[Var\left({\mathbf{y}}_B\right)\right]}^{-1}{\mathbf{g}}_{A_i,B}.\end{array} $$

Hence:

8 $$ \begin{array}{l}{r}_{A_i}=\sqrt{\frac{r_{G_{AB}}^2{\upsigma}_{a_A}^2{\upsigma}_{a_B}^2\mathbf{g}{\hbox{'}}_{A_i,B}{\left[Var\left({\mathbf{y}}_B\right)\right]}^{-1}{\mathbf{g}}_{A_i,B}}{\upsigma_{a_A}^2}}.\\ {}={r}_{G_{AB}}\sqrt{\upsigma_{a_B}^2\mathbf{g}{\hbox{'}}_{A_i,B}{\left[Var\left({\mathbf{y}}_B\right)\right]}^{-1}{\mathbf{g}}_{A_i,B}}\end{array} $$

Equation 8 contains the variance of the phenotypes of individuals from population *B*, which can be written as:

9 $$ \begin{array}{l}Var\left({\mathbf{y}}_B\right)=Cov\left({\mathbf{y}}_B,{\mathbf{y}}_B\right)\\ {}=Var\left({\mathbf{a}}_B\right)+Var\left({\mathbf{e}}_B\right)={\mathbf{G}}_B\;{\upsigma}_{a_B}^2+{\mathbf{R}}_B\;{\upsigma}_{e_B}^2,\end{array} $$

where **G**_*B*_ is the *n*_*B*_ x *n*_*B*_ genomic relationship matrix of reference individuals of population *B*, ${\upsigma}_{a_B}^2$ is the genetic variance in population *B*, **R**_*B*_ is a *n*_*B*_ x *n*_*B*_ standardized matrix that describes the correlations between environmental effects of individuals from population *B*, and ${\upsigma}_{e_B}^2$ is the environmental variance in population *B*. Substituting Equation 9 into Equation 8 results in:

10 $$ {r}_{A_i}={r}_{G_{AB}}\sqrt{\mathbf{g}{\hbox{'}}_{A_i,B}{\left[{\mathbf{G}}_B+{\mathbf{R}}_B\frac{\upsigma_{e_B}^2}{\upsigma_{a_B}^2}\right]}^{-1}{\mathbf{g}}_{A_i,B}} $$

### Deterministic accuracy of across-population genomic prediction using multiple populations in the reference population based on selection index theory

Equation 10 is valid when there is only one reference population. However, it may be interesting to combine reference populations to predict breeding values for individuals from another population. Based on a combined reference population from two populations, i.e. populations *B* and *C*, the breeding value for a selection candidate *i* of population *A* can be predicted as:

11 $$ \begin{array}{l}{\widehat{a}}_{A_i}=\left[\begin{array}{cc}\hfill \mathbf{b}{\hbox{'}}_{AB}\hfill & \hfill \mathbf{b}{\hbox{'}}_{AC}\hfill \end{array}\right]\kern0.24em \left[\begin{array}{c}\hfill {\mathbf{y}}_B\hfill \\ {}\hfill {\mathbf{y}}_C\hfill \end{array}\right]\\ {}=Cov\left({a}_{A_i},\left[\begin{array}{c}\hfill {\mathbf{y}}_B\hfill \\ {}\hfill {\mathbf{y}}_C\hfill \end{array}\right]\right)\kern0.24em {\left(Var\left[\begin{array}{c}\hfill {\mathbf{y}}_B\hfill \\ {}\hfill {\mathbf{y}}_C\hfill \end{array}\right]\right)}^{-1}\;\left[\begin{array}{c}\hfill {\mathbf{y}}_B\hfill \\ {}\hfill {\mathbf{y}}_C\hfill \end{array}\right],\end{array} $$

where **b**_*AC*_ is a *n*_*C*_ x1 vector with partial regression coefficients of breeding values of individuals from population *A* on phenotypes of population *C*, **y**_*C*_ is a *n*_*C*_ x1 vector with phenotypes corrected for fixed effects of individuals from population *C*.

Following Equation 3, the covariance between the TBV of individual *i* of population *A* and the phenotypes of individuals from populations *B* and *C* is:

12 $$ \begin{array}{l}Cov\left({a}_{A_i},\left[\begin{array}{c}\hfill {\mathbf{y}}_B\hfill \\ {}\hfill {\mathbf{y}}_C\hfill \end{array}\right]\right)\\ {}=\left[\begin{array}{cc}\hfill {r}_{G_{AB}}{\upsigma}_{a_A}{\upsigma}_{a_B}\mathbf{g}{\hbox{'}}_{A_i,B}\hfill & \hfill {r}_{G_{AC}}{\upsigma}_{a_A}{\upsigma}_{a_C}\mathbf{g}{\hbox{'}}_{A_i,C}\hfill \end{array}\right],\end{array} $$

where ${r}_{G_{AC}}$ is the genetic correlation between population *A* and population *C*, ${\upsigma}_{a_C}$ is the genetic standard deviation in population *C*, and ${\mathbf{g}}_{A_i,C}$ is a *n*_*C*_ x1 vector of genomic relationships between individual *i* of population *A* and reference individuals of population *C*.

Hence, Equation 11 can be written as:

13 $$ \begin{array}{l}{\widehat{a}}_{A_i}=\left[\begin{array}{cc}\hfill {r}_{G_{AB}}{\upsigma}_{a_A}{\upsigma}_{a_B}\mathbf{g}{\hbox{'}}_{A_i,B}\hfill & \hfill {r}_{G_{AC}}{\upsigma}_{a_A}{\upsigma}_{a_C}\mathbf{g}{\hbox{'}}_{A_i,C}\hfill \end{array}\right]\kern0.36em \times \\ {}\;{\left(Var\left[\begin{array}{c}\hfill {\mathbf{y}}_B\hfill \\ {}\hfill {\mathbf{y}}_C\hfill \end{array}\right]\right)}^{-1}\;\left[\begin{array}{c}\hfill {\mathbf{y}}_B\hfill \\ {}\hfill {\mathbf{y}}_C\hfill \end{array}\right].\end{array} $$

In this situation, Equation 6 can also be used to calculate the accuracy. The covariance between the predicted and TBV of individual *i* of population *A* based on a reference population of individuals from populations *B* and *C* is:

14 $$ \begin{array}{l}Cov\left({\widehat{a}}_{A_i},{a}_{A_i}\right)\\ {}=Cov\left(\left[\begin{array}{cc}\hfill {r}_{G_{AB}}{\upsigma}_{a_A}{\upsigma}_{a_B}\mathbf{g}{\hbox{'}}_{A_i,B}\hfill & \hfill {r}_{G_{AC}}{\upsigma}_{a_A}{\upsigma}_{a_C}\mathbf{g}{\hbox{'}}_{A_i,C}\hfill \end{array}\right]\kern0.48em \times \right.\\ {}\;\left.\;{\left(Var\left[\begin{array}{c}\hfill {\mathbf{y}}_B\hfill \\ {}\hfill {\mathbf{y}}_C\hfill \end{array}\right]\right)}^{-1}\kern0.24em \left[\begin{array}{c}\hfill {\mathbf{y}}_B\hfill \\ {}\hfill {\mathbf{y}}_C\hfill \end{array}\right],{a}_{A_i}\right)\\ {}=\left[\begin{array}{cc}\hfill {r}_{G_{AB}}{\upsigma}_{a_A}{\upsigma}_{a_B}\mathbf{g}{\hbox{'}}_{A_i,B}\hfill & \hfill {r}_{G_{AC}}{\upsigma}_{a_A}{\upsigma}_{a_C}\mathbf{g}{\hbox{'}}_{A_i,C}\hfill \end{array}\right]\times \\ {}\;{\left(Var\left[\begin{array}{c}\hfill {\mathbf{y}}_B\hfill \\ {}\hfill {\mathbf{y}}_C\hfill \end{array}\right]\right)}^{-1}\kern0.36em \left[\begin{array}{c}\hfill {r}_{G_{AB}}{\upsigma}_{a_A}{\upsigma}_{a_B}{\mathbf{g}}_{A_i,B}\hfill \\ {}\hfill {r}_{G_{AC}}{\upsigma}_{a_A}{\upsigma}_{a_C}{\mathbf{g}}_{A_i,C}\hfill \end{array}\right].\end{array} $$

Using this expression in Equation 6, the accuracy of genomic prediction becomes:

15 $$ \begin{array}{l}{r}_{A_i}=\sqrt{\left[\begin{array}{cc}\hfill {r}_{G_{AB}}{\upsigma}_{a_B}\mathbf{g}{\hbox{'}}_{A_i,B}\hfill & \hfill {r}_{G_{AC}}{\upsigma}_{a_C}\mathbf{g}{\hbox{'}}_{A_i,C}\hfill \end{array}\right]\times}\\ {}\;\overline{\;{\left(Var\;\left[\begin{array}{c}\hfill {\mathbf{y}}_B\hfill \\ {}\hfill {\mathbf{y}}_C\hfill \end{array}\right]\right)}^{-1}\kern0.36em \left[\begin{array}{c}\hfill {r}_{G_{AB}}{\upsigma}_{a_B}{\mathbf{g}}_{A_i,B}\hfill \\ {}\hfill {r}_{G_{AC}}{\upsigma}_{a_C}{\mathbf{g}}_{A_i,C}\hfill \end{array}\right]}.\end{array} $$

The (co-)variances of the phenotypes of the reference individuals of populations *B* and *C* in Equation 15 can be written as:

16 $$ Var\;\left[\begin{array}{c}\hfill {\mathbf{y}}_B\hfill \\ {}\hfill {\mathbf{y}}_C\hfill \end{array}\right]=\left[\begin{array}{cc}\hfill Var\left({\mathbf{y}}_B\right)\hfill & \hfill Cov\left({\mathbf{y}}_B,{\mathbf{y}}_C\right)\hfill \\ {}\hfill Cov\hbox{'}\left({\mathbf{y}}_B,{\mathbf{y}}_C\right)\hfill & \hfill Var\left({\mathbf{y}}_C\right)\hfill \end{array}\right] $$

The variance of the phenotypes within one population follows from Equation 9. The covariance of the phenotypes across the two populations is:

17 $$ \begin{array}{l}Cov\left({\mathbf{y}}_B,{\mathbf{y}}_C\right)=Cov\left({\mathbf{a}}_B+{\mathbf{e}}_B,\;{\mathbf{a}}_C+{\mathbf{e}}_C\right)\\ {}=Cov\left({\mathbf{a}}_B,{\mathbf{a}}_C\right)={r}_{G_{BC}}{\upsigma}_{a_B}{\upsigma}_{a_C}{\mathbf{G}}_{BC}\;.\end{array} $$

Combining Equations 9, 16, and 17 yields:

18 $$ Var\left[\begin{array}{c}\hfill {\mathbf{y}}_B\hfill \\ {}\hfill {\mathbf{y}}_C\hfill \end{array}\right]=\left[\begin{array}{cc}\hfill {\mathbf{G}}_B{\upsigma}_{a_B}^2+{\mathbf{R}}_B{\upsigma}_{e_B}^2\hfill & \hfill {r}_{G_{BC}}{\upsigma}_{a_B}{\upsigma}_{a_C}{\mathbf{G}}_{BC}\hfill \\ {}\hfill {r}_{G_{BC}}{\upsigma}_{a_B}{\upsigma}_{a_C}{\mathbf{G}}_{BC}^{\hbox{'}}\hfill & \hfill {\mathbf{G}}_C{\upsigma}_{a_C}^2+{\mathbf{R}}_C{\upsigma}_{e_C}^2\hfill \end{array}\right]. $$

Substituting this result into Equation 15 yields:

19 $$ \begin{array}{l}{r}_{A_i}=\sqrt{\left[\begin{array}{cc}\hfill {r}_{G_{AB}}{\upsigma}_{a_B}\mathbf{g}{\hbox{'}}_{A_i,B}\hfill & \hfill {r}_{G_{AC}}{\upsigma}_{a_C}\mathbf{g}{\hbox{'}}_{A_i,C}\hfill \end{array}\right]\times {\left[\begin{array}{cc}\hfill {\mathbf{G}}_B{\upsigma}_{a_B}^2+{\mathbf{R}}_B{\upsigma}_{e_B}^2\hfill & \hfill {r}_{G_{BC}}{\upsigma}_{a_B}{\upsigma}_{a_C}{\mathbf{G}}_{BC}\hfill \\ {}\hfill {r}_{G_{BC}}{\upsigma}_{a_B}{\upsigma}_{a_C}{\mathbf{G}}_{BC}^{\hbox{'}}\hfill & \hfill {\mathbf{G}}_C{\upsigma}_{a_C}^2+{\mathbf{R}}_C{\upsigma}_{e_C}^2\hfill \end{array}\right]}^{-1}\times \left[\begin{array}{c}\hfill {r}_{G_{AB}}{\upsigma}_{a_B}{\mathbf{g}}_{A_i,B}\hfill \\ {}\hfill {r}_{G_{AC}}{\upsigma}_{a_C}{\mathbf{g}}_{A_i,C}\hfill \end{array}\right]}\\ {}=\sqrt{\left[\begin{array}{cc}\hfill {r}_{G_{AB}}\mathbf{g}{\hbox{'}}_{A_i,B}\hfill & \hfill {r}_{G_{AC}}\mathbf{g}{\hbox{'}}_{A_i,C}\hfill \end{array}\right]\times {\left[\begin{array}{cc}\hfill {\mathbf{G}}_B+{\mathbf{R}}_B\frac{\upsigma_{e_B}^2}{\upsigma_{a_B}^2}\hfill & \hfill {r}_{G_{BC}}{\mathbf{G}}_{BC}\hfill \\ {}\hfill {r}_{G_{BC}}{\mathbf{G}}_{BC}^{\hbox{'}}\hfill & \hfill {\mathbf{G}}_C+{\mathbf{R}}_C\frac{\upsigma_{e_C}^2}{\upsigma_{a_C}^2}\hfill \end{array}\right]}^{-1}\times \left[\begin{array}{c}\hfill {r}_{G_{AB}}{\mathbf{g}}_{A_i,B}\hfill \\ {}\hfill {r}_{G_{AC}}{\mathbf{g}}_{A_i,C}\hfill \end{array}\right]}.\end{array} $$

Although Equation 19 is derived for across-population genomic prediction, this formula can also be applied to estimate the accuracy of multi population genomic prediction for which one of the reference populations is the population of the selection candidates. Moreover, it is interesting to note that when one population is included in the reference population and selection candidates are from the same population as the reference individuals, Equation 19 becomes equivalent to the expression derived by VanRaden [2].

### Deterministic accuracy of across-population genomic prediction based on population parameters

In general, the accuracy with which an effect is predicted equals the square root of the proportion of variance explained by the effect. The accuracy of a sire’s EBV based on progeny information, for example, equals $$ \sqrt{\frac{{\scriptscriptstyle \raisebox{1ex}{$1$}\!\left/ \!\raisebox{-1ex}{$4$}\right.}{\upsigma}_a^2}{{\scriptscriptstyle \raisebox{1ex}{$1$}\!\left/ \!\raisebox{-1ex}{$4$}\right.}{\upsigma}_a^2+\left({\upsigma}_p^2-\raisebox{1ex}{$1$}\!\left/ \!\raisebox{-1ex}{$4$}\right.{\upsigma}_a^2\right)/n}} $$, where the numerator is the variance due to the sire, and the denominator the variance of the average of *n* progeny [20]. In the same way, when each chromosome segment explains an amount of variance equal to $$ {\upsigma}_a^2/{M}_e $$, in which *M*_*e*_ is the effective number of chromosome segments [25], the accuracy of the predicted segment effect equals:

20 $$ r=\sqrt{\frac{\upsigma_a^2/{M}_e}{\upsigma_a^2/{M}_e+{\upsigma}_p^2/{N}_p}}, $$

where ${\upsigma}_p^2$ is the phenotypic variance and *N*_*p*_ is the size of the reference population. In the denominator, it is assumed that a single segment explains very little variance, so that ${\upsigma}_p^2-{\upsigma}_a^2/{M}_e\approx {\upsigma}_p^2$. When the accuracy is the same for all effective segments, this is also the accuracy of genomic prediction. Multiplying both numerator and denominator of Equation 20 by ${N}_p{M}_e/{\upsigma}_p^2$ yields a simple expression for the accuracy of genomic prediction for all selection candidates of the same population:

21 $$ {r}_P=\sqrt{\frac{N_p{h}^2}{N_p{h}^2+{M}_e}}, $$

where *h*^2^ is the heritability of the trait. This result was originally derived by Daetwyler *et al.* [1,16], but with a more complex derivation.

For within-population genomic prediction, *M*_*e*_ follows from [25]:

22 $$ {M}_e=\frac{1}{Var\left({\mathbf{G}}_{R{P}_{ij}}-{\mathbf{A}}_{R{P}_{ij}}\right)}, $$

where ${\mathbf{G}}_{R{P}_{ij}}$ is the genomic relationship between individuals *i* and *j* from the reference population, ${\mathbf{A}}_{R{P}_{ij}}$ is the corresponding pedigree relationship, and the variance is taken over all pairs *ij* in the reference population. For across-population genomic prediction, we propose the following analogy:

23 $$ {M}_e=\frac{1}{Var\left({\mathbf{G}}_{R{P}_i,S{K}_j}-{\mathbf{A}}_{R{P}_i,S{K}_j}\right)}, $$

in which the index *RP*_*i*_,*SK*_*j*_ refers to reference individual *i* and selection candidate *j*, and the variance is taken over all the pair-wise relationships between reference individuals and selection candidates. As explained by Goddard *et al.* [25], the expectation of the genomic relationships for unrelated animals should be 0. This can be achieved by using population-specific allele frequencies to rescale the genotypes for setting up ${\mathbf{G}}_{R{P}_i,S{K}_j}$, as explained before for the expression based on selection index theory.

For across-population genomic prediction, the genetic correlation between populations has to be taken into account, because it limits the part of the genetic variance in the selection candidates that can be explained by the reference population. Therefore, the genetic correlation between the reference population and the selection candidates, ${r}_{G_{RP,SK}}$, was incorporated into Equation 21, giving:

24 $$ {r}_P={r}_{G_{RP,SK}}\sqrt{\frac{N_p{h}^2}{N_p{h}^2+{M}_e}}. $$

### Simulations

#### Genotypes

Genotypes were available for 1285 dairy cows from the Netherlands that originated from three breeds (1033 Holstein-Friesian (HF), 105 Groninger White Headed (GWH), and 147 Meuse-Rhine-Yssel (MRY)). All individuals were pure-bred animals since at least 87.5% of their genes originated from one of the three breeds.

Individuals from the breeds GWH and MRY were genotyped with the Illumina BovineHD Beadchip (777 k, Illumina, San Diego, CA). Quality controls consisted in removing genotypes with a GenCall (GC) score lower than 0.2, SNPs with a call rate smaller than 95% in one of the breeds and SNPs with an unknown map position or located on the sex chromosomes. The HF individuals were genotyped with the Illumina BovineSNP50 Beadchip (50 k, Illumina, San Diego, CA), and imputed to high-density (777 k) using a reference population of 3150 HF individuals as described by Pryce *et al.* [26]. Quality control consisted in removing SNPs with a call rate smaller than 95% or with an unknown map position or located on the sex chromosomes. After editing the imputed genotypes, the mean Beagle *R*^2^ value, which reflects the accuracy of imputation, was equal to 0.96 across imputed loci, which indicates that imputation was highly accurate.

Loci for which the genotypes passed the quality control of both the HF dataset and the combined GWH and MRY dataset were retained in the entire dataset. From this entire dataset, SNPs with a minor allele frequency equal to or lower than 0.5%, SNPs for which only two genotypes were observed, and SNPs in complete LD (r^2^ = 1) with an adjacent SNP were removed. To increase the power of accurately estimating genomic breeding values, arbitrarily, we took only three chromosomes, namely chromosomes 13, 23 and 28 that contained about 10% of the remaining high-density SNPs into account. According to the literature, the LD pattern of those chromosomes is comparable to the LD pattern of the entire cattle genome [27,28]. After editing, a total of 31 503 SNPs remained across the three chromosomes.

#### Simulation of phenotypes

Phenotypes of the individuals were simulated using different scenarios with two variables i.e. (1) the number of QTL underlying the simulated trait and (2) the correlation between allele substitution effects of the QTL underlying the simulated trait in the different populations, i.e. the genetic correlation between populations [20,23]. From the 31 503 SNPs available after editing, 5000 were randomly selected to become candidate QTL, regardless of the chromosome. In each replicate, the actual QTL with an effect on the trait were randomly sampled from those candidate QTL. The remaining (31 503 – 5000 =) 26 503 SNPs composed the group of markers used in all analyses. Using this approach allowed us to keep the set of markers constant across all replicates but still made it possible to randomly select the QTL from the group of candidate QTL within each replicate. The numbers of QTL underlying the simulated trait were equal to 3000 (~10% of all SNPs), 300 (~1%), 30 (~0.1%) or 3 (~0.01%).

The allele substitution effects of QTL were sampled from a multinormal distribution with mean 0 and standard deviation 1, assuming a correlation of 1, 0.8, 0.6, 0.4, or 0.2 between the allele substitution effects across all three pairs of breeds. This was simulated by sampling random numbers from a normal distribution with mean 0 and standard deviation 1 and multiplying those numbers with the Cholesky decomposition of the covariance matrix between the allele substitution effects of the breeds.

For each of the individuals, the TBV was calculated by multiplying the simulated allele substitution effects with the genotypes of the 3000, 300, 30, or 3 QTL coded as 0, 1, and 2. Only additive effects and no dominance effects or epistatic interactions were simulated, therefore, the effects were summed over all QTL. Finally, TBV of all individuals of the three breeds were rescaled to a mean of 0 and variance of 1 across breeds. By rescaling the TBV in this way, their mean and variance were the same for each replicate and for the different numbers of QTL, which indicates that when the number of QTL was higher, each QTL explained a smaller part of the variance.

Allele frequencies for simulated QTL (sampled from the SNPs) differed for each of the three breeds, resulting in differences in average TBV between the breeds. To simulate environmental effects for each individual assuming equal heritability for the three breeds, TBV were first adjusted by subtracting the average TBV of the individual’s breed before the genetic variance across TBV was calculated. Thereafter, the environmental effect per individual was sampled for the three breeds from a normal distribution with mean 0 and variance $\left(\frac{1}{h^2}-1\right)$*(variance of TBV corrected for mean TBV within breed). For each individual, the phenotype was calculated as the sum of its TBV and the randomly sampled environmental effect. Note that the within-breed TBV means were only subtracted from the TBV to calculate the environmental variance, the TBV itself, and therefore the phenotypes as well, still included the within-breed TBV mean.

For each scenario, simulations were replicated 100 times using a heritability of 0.95 to simulate phenotypes in each of the three breeds and for each number of QTL underlying the trait. A high heritability of 0.95 was chosen to increase the achieved accuracies and to make the differences in accuracies between the different scenarios more pronounced for the size of reference population used. In dairy cattle breeding, a heritability of 0.95 can be achieved by using deregressed proofs of bulls for a trait with a heritability of 0.25 based on 285 daughters, following [29]:

$$ r=\sqrt{\frac{n{h}^2}{n{h}^2+\left(4-{h}^2\right)}}, $$

where *r* is the accuracy for a sire’s breeding value, *n* is the number of daughters of a sire, and *h*^2^ is the heritability of the trait.

#### Scenarios to evaluate accuracy of genomic prediction

Mean accuracy of genomic prediction was empirically and deterministically evaluated for five different scenarios. The first scenario, i.e. the base scenario, which represented single-population genomic prediction, used HF animals as reference population and selection candidates. In the other scenarios, the reference population consisted of one or two populations and breeding values were predicted for individuals from another population, which means that across-population genomic prediction was applied (Table 1). For the across-population scenarios, the reference population was the same for all selection candidates of a specific population. In the scenario with HF individuals both as reference population and selection candidates, the deterministic accuracies (Equations 19 and 24) were calculated for a single HF individual using a reference population consisting of all remaining HF individuals. The empirical accuracy was calculated using 20-fold cross-validation, where in each replicate, individuals were randomly divided in 20 equally-sized groups using each group once as selection candidates and the remaining 19 as reference population.

**Table 1:** **Overview of the breeds used in the different reference populations and as selection candidates**

	**Reference population**		**Predicted individuals**	
**Scenario**	**Breed(s)**	**Nb of individuals**	**Breed**	**Nb of individuals**
**Base**	HF	1032/981-982^1^	HF	1/51-52^1^
**1**	HF	1033	GWH	105
**2**	HF + MRY	1180	GWH	105
**3**	HF	1033	MRY	147
**4**	HF + GWH	1138	MRY	147

^1^Deterministic formulas used leave-one-out cross-validation, empirical calculations used 20 fold cross-validation using 20 groups of 51 or 52 individuals due to computational reasons; HF = Holstein-Friesian; MRY = Meuse-Rhine-Yssel; GWH = Groninger White Headed.

#### Empirical accuracy based on simulated phenotypes

For the empirical estimation of the accuracy, a GBLUP–model type, called GREML, was run in ASReml [30]. This GREML model used a genomic relationship matrix (**G**) and simulated phenotypes based on 3000, 300, 30 or 3 QTL underlying the simulated trait. In this model, breed was included as a fixed effect. This model is termed GREML, because it has the same features as the commonly known GBLUP model, however variances were not assumed to be known but were estimated simultaneously with the breeding values using REML. Accuracy was calculated for each population as the correlation between EBV from this model and TBV. Since simulated phenotypes were different per replicate, averages and standard errors of empirical accuracies were calculated across replicates.

The **G** matrix used in GREML contained all reference individuals and selection candidates and was calculated based on the method of Yang *et al.* [24]; ${\mathbf{G}}_{SNPs}=\frac{\mathbf{XX}\hbox{'}}{n}$. In this equation, *n* represents the number of SNPs (26 503) and the **X** matrix contains standardized genotypes (one locus per column) of each individual (one individual per row). For the empirical estimation of the accuracy, standardized genotypes were calculated as ${x}_{ij}=\frac{g_{ij}-2{p}_j}{\sqrt{2{p}_j\left(1-{p}_j\right)}}$, where *g*_*ij*_ codes the genotype for individual *i* at marker locus *j* as 0, 1 and 2, and *p*_*j*_ is the allele frequency at marker locus *j* for the second allele averaged over all breeds. To calculate the average allele frequency per locus, the allele frequency per locus was calculated per breed and thereafter averaged over the three breeds, with an equal weight for each of the breeds. In that way, average allele frequency is not dominated by the breed with the largest number of genotyped individuals. Note that for each scenario, the **G**_*SNPs*_ matrix contained only the reference individuals and selection candidates (and the SNPs segregating in that group), so four different **G**_*SNPs*_ matrices were calculated that contained (1) all HF individuals (26 486 SNPs), (2) all HF and GWH individuals (26 500 SNPs), (3) all HF and MRY individuals (26 498 SNPs), and (4) all HF, GWH and MRY individuals (26 503 SNPs).

In the calculation of **G**_*SNPs*_, allele frequencies of the current population were used, which means that the current population was used as the base population. This indicates that the inbreeding level in **G**_*SNPs*_ differed from the inbreeding level in the pedigree-based relationship matrix, **A**, and that **G**_*SNPs*_ and **A** were not compatible. To rescale the inbreeding level in **G**_*SNPs*_ to the inbreeding level of **A**, the following adjustment was made to within-breed genomic relationships [31]:

$$ {\mathbf{G}}_{SNPs}^{*}=\left(1-\overline{F_b}\right)\;{\mathbf{G}}_{SNPs}+2\overline{F_b}\;\mathbf{J}, $$

where *F*_*b*_ was the average inbreeding coefficient of all individuals of breed *b* based on the pedigree and **J** was a matrix filled with ones.

Due to only three chromosomes being selected for this study and due to sampling variance of the SNPs on the chip, $E\left(\mathbf{G}\Big|{\mathbf{G}}_{SNPs}^{*}\right)$ is not ${\mathbf{G}}_{SNPs}^{*}$ [25,31]. Therefore, we regressed the ${\mathbf{G}}_{SNPs}^{*}$ matrix back to the **A** matrix, which is the additive genetic relationship matrix based on the pedigree, following Yang *et al.* [24] and Goddard *et al.* [25]:

$$ \widehat{\mathbf{G}}=\mathbf{A}+b\left({\mathbf{G}}_{SNPs}^{*}-\mathbf{A}\right), $$

where

$$ b=\frac{Var\left({\mathbf{G}}_{SNPs}^{*}-\mathbf{A}\right)}{\left[Var\left({\mathbf{G}}_{SNPs}^{*}-\mathbf{A}\right)\right]+Var\left(\mathbf{E}\right)}=\frac{Var\left(\widehat{\mathbf{G}}-\mathbf{A}\right)-1/n}{Var\left(\widehat{\mathbf{G}}-\mathbf{A}\right)}. $$

Since the level of family relationships influences the sampling error on the elements in **G**, the regression coefficient *b* was calculated separately for bins of family relationships in **A** (0–0.10, > 0.10-0.25, > 0.25-0.50 and > 0.5) within each breed and for each combination of breeds. Across-breed relationships were indeed 0 in **A**, so in that case *Var*(**Ĝ** − **A**) approximately reduced to *Var*(**Ĝ**). Parent-offspring relationships and self-relationships were not or hardly affected by sampling error and therefore excluded from the regression. The regression coefficient *b* was always above 0.95, and, in most cases, even above 0.99. Therefore, the effect of regressing the **G** matrix back to the **A** matrix was limited.

The inbreeding level in **A** depends on the depth of the pedigree, which indicates that different pedigree depths across populations can cause differences in inbreeding levels across the populations. To remove these differences in pedigree depth, the pedigree was cut off at seven generations for all individuals. Based on the pedigree, small relationships between some animals of the different breeds occurred, with a maximum relationship of 0.035 between HF and GWH, 0.034 between HF and MRY, and 0.029 between GWH and MRY. These relationships resemble more or less the relationship between an individual and one of its ancestors five generations back.

#### Deterministic accuracies of genomic prediction

For each scenario, accuracies of genomic prediction were deterministically derived using the two methods explained before; one method based on selection index theory (Equation 19) and one method based on population parameters (Equation 24). It is interesting to note that the formula based on selection index theory provides a single accuracy for each selection candidate, while the formula using population parameters provides an accuracy that applies to all selection candidates of the same population. Both deterministic methods calculate the accuracy based on genomic relationships and do not use phenotypes. Since the subset of SNPs was constant across all replicates and scenarios with different numbers of QTL, only one accuracy was calculated that applied to all replicates and numbers of QTL. Therefore, it was not possible to calculate standard errors across replicates for the deterministic accuracies.

#### Estimating genetic correlations between populations

In this simulation study, the genetic correlation between populations was known. In studies using real data, this is usually not the case and the genetic correlation needs to be estimated from the data. We investigated how accurate the genetic correlations between HF and GWH, and between HF and MRY were when using a multi-trait model in ASReml [30] in which the same trait in different breeds was treated as different traits. Within the multi-trait model, the same **G** matrix was used as in the GBLUP model, the environmental correlation was set to 0 and genetic and environmental variances of GWH and MRY animals were fixed at the simulated values, because the small number of animals in those breeds made it difficult to estimate variance components reliably.

## Results

### Differences between populations

In this study, accuracy of genomic prediction was evaluated by using genotypes of three cattle breeds. In cases where allele substitution effects were equal across breeds, differences in accuracy between single- and across-breed genomic predictions were due to differences in allele frequencies, relationships and LD pattern across breeds. The correlation between allele frequencies of all 26 503 SNPs was 0.67 for HF and GWH, 0.73 for HF and MRY, and 0.65 for GWH and MRY. Correlations of allele frequencies of SNPs and candidate QTL across breeds were similar.

Based on pedigree information, there were few differences in average relationships between breeds with average relationships of 0.0004 between HF and GWH (ranging from 0 to 0.035, 0.0004 between HF and MRY (ranging from 0 to 0.034), and 0.0005 between GWH and MRY (ranging from 0 to 0.029). Based on genotype data, differences in average relationships across breeds became more pronounced, with average relationships of −0.084 between HF and GWH (ranging from −0.194 to +0.115), −0.050 between HF and MRY (ranging from −0.151 to +0.125), and −0.098 between GWH and MRY (ranging from −0.184 to +0.088).

### Equal allele substitution effects across populations

**Figure 1:**
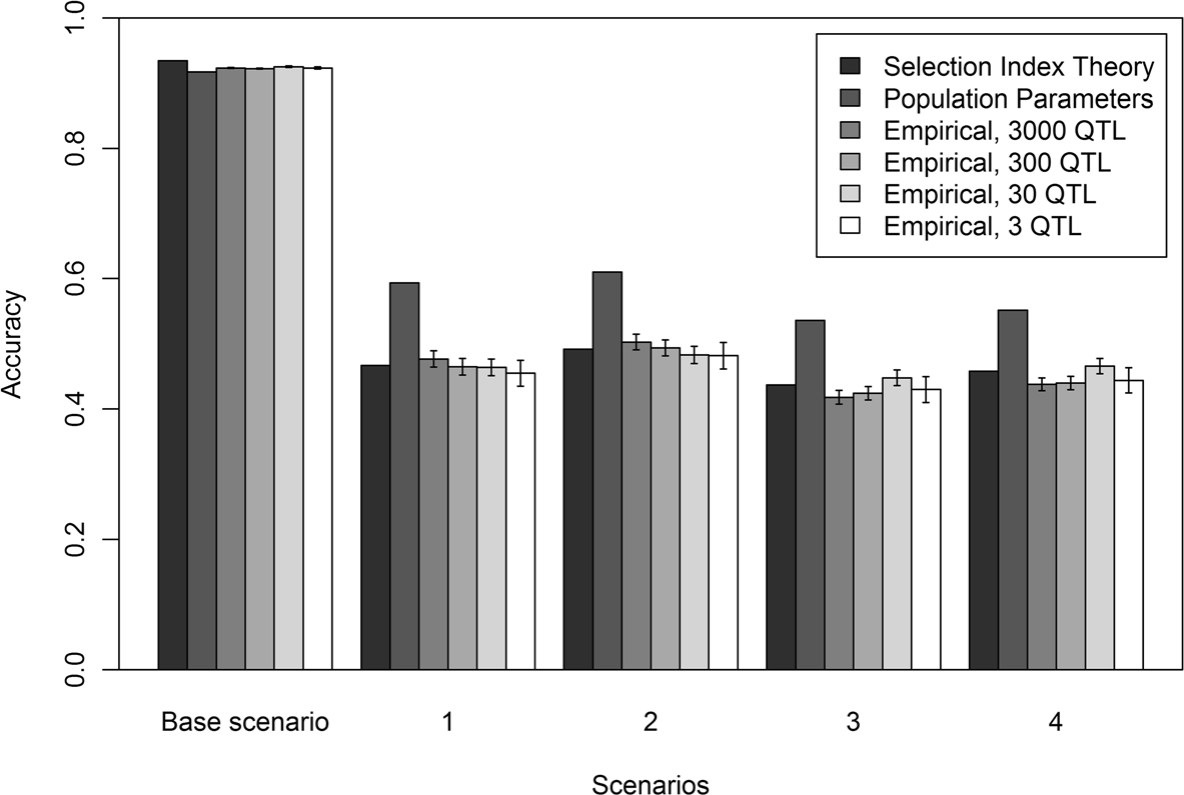
**Empirical and deterministic accuracies of genomic prediction with a genetic correlation of 1.** Empirical and deterministic accuracies of genomic prediction (± standard error) with a heritability of 0.95 using equal allele substitution effects of the QTL underlying the simulated trait in the three breeds for five different scenarios; Base = reference HF (Holstein-Friesian) population, selection candidates HF; 1 = reference population HF, selection candidates GWH (Groninger White Headed); 2 = reference population HF and MRY (Meuse-Rhine-Yssel), selection candidates GWH; 3 = reference population HF, selection candidates MRY; 4 = reference population HF and GWH, selection candidates MRY.

Accuracies of genomic prediction are in Figure 1 for scenarios with equal allele substitution effects for the three breeds. Figure 1 shows that standard errors for all empirically calculated accuracies were small. Since both deterministic accuracies did not use replicates, there are no standard errors across replicates. However, the method based on selection index theory estimates accuracy per individual and this accuracy depended on the relationships of the selection candidate with the reference individuals. For each scenario, standard errors of the accuracy were calculated over all individuals and were equal to (mean and standard errors) 0.934 ± 0.001 (base scenario), 0.467 ± 0.006 (scenario 1), 0.492 ± 0.006 (scenario 2), 0.437 ± 0.003 (scenario 3), and 0.458 ± 0.003 (scenario 4).

Accuracies for the base scenario, for which breeding values of HF individuals were predicted using a reference population of HF individuals, were very high (>0.9). Empirically derived accuracies were the same for the different numbers of QTL underlying the trait, which indicates that the number of QTL did not affect empirical accuracy in single-breed genomic prediction. With both deterministic methods, accuracies were in good agreement with the empirically-derived accuracies.

Accuracies with the other four scenarios, for which across-breed genomic prediction was applied, were much lower than those with the base scenario, but still ranged from 0.4 to 0.5. In each scenario, empirical accuracies using different numbers of QTL underlying the trait were very similar, which indicates that there is no effect of number of QTL on empirical accuracy. As with single-breed genomic prediction, estimated accuracies based on selection index theory were in good agreement with empirical accuracies for all four scenarios of across-breed genomic prediction. The deterministic prediction formula using population parameters overestimated empirical accuracies by about 25%.

Empirical accuracies as well as deterministic accuracies were slightly higher for selection candidates from breed GWH than for those from breed MRY. For both breeds, empirical and deterministic accuracies slightly increased when the other breed was added to the HF reference population, thus maintaining a near constant difference in accuracy between GWH and MRY individuals.

### Different allele substitution effects across populations

**Figure 2:**
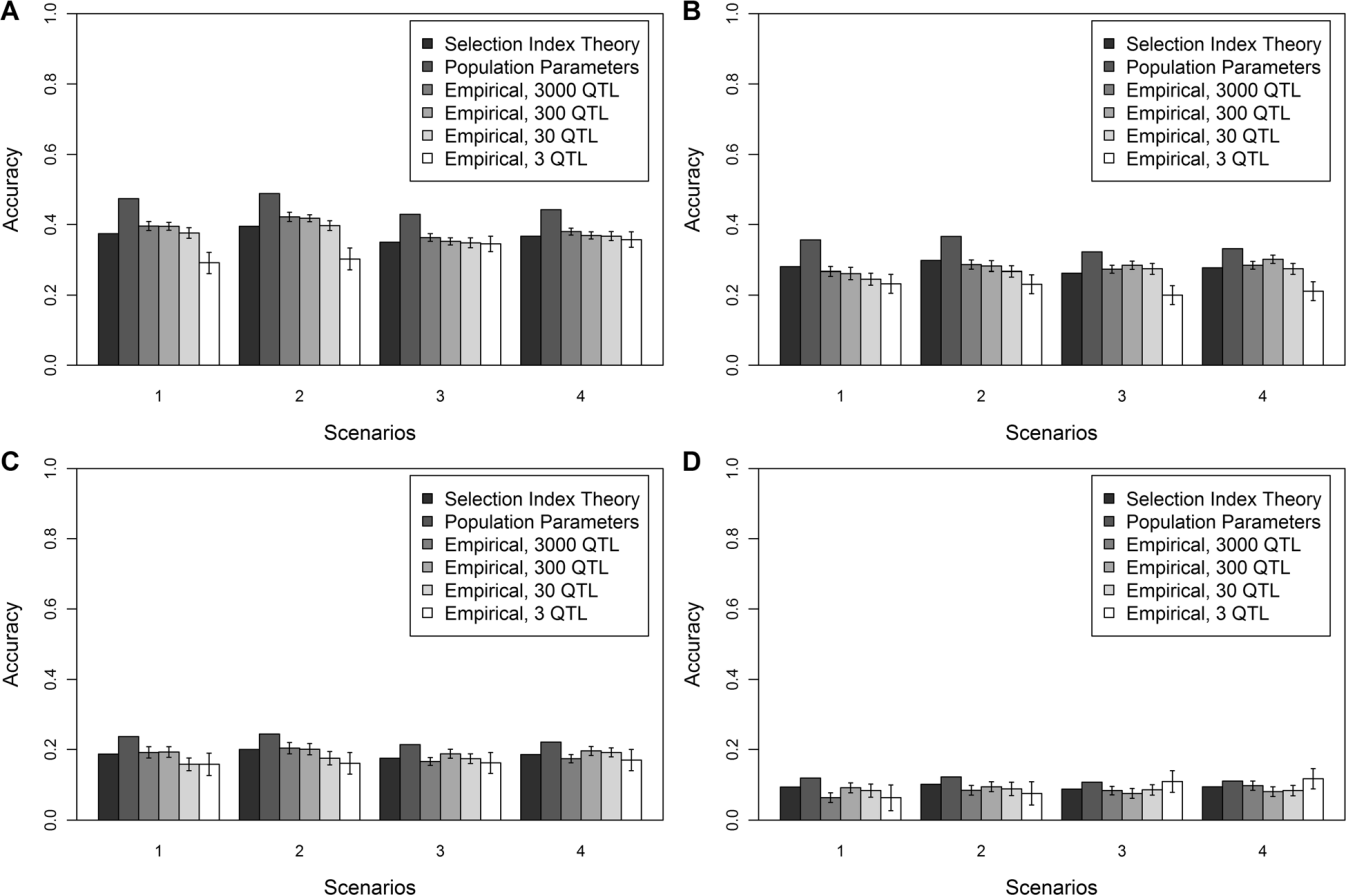
**Empirical and deterministic accuracies of genomic prediction at different genetic correlations.** Empirical and deterministic accuracies of genomic prediction (± standard error) at a heritability of 0.95 using a correlation of **(A)** 0.8, **(B)** 0.6, **(C)** 0.4, or **(D)** 0.2 between allele substitution effects of the QTL underlying the simulated trait in the different breeds for four different scenarios. 1 = reference population HF (Holstein-Friesian), selection candidates GWH (Groninger White Headed); 2 = reference population HF and MRY (Meuse-Rhine-Yssel), selection candidates GWH; 3 = reference population HF, selection candidates MRY; 4 = reference population HF and GWH, selection candidates MRY.

Accuracies of genomic prediction are in Figure 2 for scenarios with a correlation of allele substitution effects across breeds equal to (A) 0.8, (B) 0.6, (C) 0.4, or (D) 0.2. Standard errors for the empirical accuracies were low as with scenarios with equal allele substitution effects across breeds. The average estimated accuracies based on selection index theory and the variances across all individuals decreased for each scenario, the reduction being proportional to the correlation between allele substitution effects across populations.

As expected, deterministic and empirical accuracies were about equal to the accuracies obtained with equal allele substitution effects across breeds multiplied by the correlation between allele substitution effects. Empirical accuracies across the different numbers of QTL underlying the trait were again very similar, although those obtained with the 3-QTL scenario seemed to differ slightly from the other scenarios. This is in agreement with the much higher standard error across the replicates obtained with the 3–QTL scenario than with the 3000-, 300- or 30-QTL scenarios.

As in scenarios with equal allele substitution effects across breeds, accuracies obtained with the formula based on selection index theory were in good agreement with empirical accuracies. This indicates that this formula can be used to estimate the accuracy even when the genetic correlation between populations differs from 1. The formula using population parameters overestimated empirical accuracies by about 25% to 30%, regardless of the genetic correlation between breeds.

### Estimated genetic correlations between populations

Estimated genetic correlations are in Table 2 for the different scenarios. When the simulated genetic correlation was 1, the genetic correlations between the breeds were slightly underestimated and ranged from 0.85 to 0.92. When the simulated genetic correlation was different from 1, estimated and simulated genetic correlations between the breeds were in good agreement for the 3000-, 300- and 30-QTL scenarios. The estimated genetic correlation for the 3-QTL scenario was generally much lower than the simulated value, which is in agreement with the results found for the empirical accuracies and is probably due to the higher sampling error on the correlation in this scenario.

**Table 2:** **Simulated and estimated genetic correlations (standard errors across replicates) between the populations**

	**Simulated genetic correlation**	**Estimated genetic correlation (s.e.)**							
**Populations**		**3000 QTL**		**300 QTL**		**30 QTL**		**3 QTL**	
**HF - GWH**	**1.0**	0.913	(0.012)	0.915	(0.012)	0.889	(0.014)	0.860	(0.022)
**HF - GWH**	**0.8**	0.791	(0.016)	0.785	(0.014)	0.769	(0.021)	0.555	(0.049)
**HF - GWH**	**0.6**	0.605	(0.022)	0.601	(0.023)	0.568	(0.027)	0.527	(0.053)
**HF - GWH**	**0.4**	0.472	(0.024)	0.507	(0.026)	0.440	(0.031)	0.309	(0.061)
**HF - GWH**	**0.2**	0.194	(0.028)	0.218	(0.027)	0.203	(0.039)	0.156	(0.067)
**HF - MRY**	**1.0**	0.888	(0.013)	0.893	(0.012)	0.911	(0.012)	0.851	(0.024)
**HF - MRY**	**0.8**	0.806	(0.017)	0.778	(0.016)	0.806	(0.016)	0.690	(0.039)
**HF - MRY**	**0.6**	0.614	(0.022)	0.688	(0.018)	0.616	(0.024)	0.456	(0.054)
**HF - MRY**	**0.4**	0.440	(0.022)	0.450	(0.025)	0.444	(0.028)	0.281	(0.062)
**HF - MRY**	**0.2**	0.244	(0.024)	0.253	(0.025)	0.239	(0.038)	0.235	(0.061)

HF = Holstein-Friesian; MRY = Meuse-Rhine-Yssel; GWH = Groninger White Headed.

## Discussion

### Deterministic accuracy of across-population genomic prediction

The first objective of this study was to develop a deterministic formula to investigate the accuracy of across-population genomic prediction. Our study as other previous studies [2,18,32] shows that the formula based on selection index theory (Equation 19) and the formula using population parameters (Equation 24) can accurately estimate the accuracy of genomic prediction within one population using relationship matrices. By setting up across-population genomic relationship matrices based on population-specific allele frequencies, it was also possible to accurately estimate the accuracy of across-population genomic prediction based on selection index theory. The application of the prediction formula using population parameters, as described in our study, overestimated the empirical accuracy for across-population genomic prediction in all scenarios by about 25 to 30%.

The genetic correlation in the deterministic formulas accounts for differences in allele substitution effects across populations. These differences may also lead to differences in genetic variances across populations, i.e. heterogeneous variances. For example, among populations, the genetic variance tends to be larger for the population with the highest mean for a given trait [33,34]. In addition, differences in allele frequencies across populations may also lead to heterogeneous variances; for example, a QTL may only segregate in one of the populations, which results in differences in the genetic variance explained by that QTL across populations although the actual allele substitution effects could be the same. Moreover, environmental variances may be different across populations when deregressed proofs of bulls are used as phenotypes, since the heritability of those proofs depends on the number of daughters of the bull, which can differ across populations. Heterogeneous variances across populations, which are not properly accounted for, may affect bias and accuracy of EBV. The deterministic formula based on selection index theory can take those heterogeneous variances into account as well, in contrast to the application of the formula based on populations parameters described here. Makgahlela *et al.* [35] empirically showed that accuracies of multi-breed genomic prediction can be increased by accounting for those heterogeneous variances across breeds in a multi-trait random regression model [35,36].

The genomic relationship matrix used in the deterministic formulas was calculated based on population-specific allele frequencies. Harris and Johnson [37] already mentioned that differences in allele frequencies should be taken into account to calculate genomic covariances and relationships between individuals of different populations. Not using population-specific allele frequencies results in average genomic relationships across populations different from 0 [38], large differences in average diagonal elements across populations [12,37] and overestimation of the accuracies [9]. In our study, using population-specific allele frequencies resulted in average genomic relationship close to 0, i.e. equal to 0.00003 with a standard deviation of 0.023 between HF and GWH, and 0.00003 with a standard deviation of 0.020 between HF and MRY.

The deterministic formula based on selection index theory (Equation 19) estimated the accuracy of across-population genomic prediction accurately for all scenarios. With a genetic correlation of 0.8, 0.6, 0.4, or 0.2, empirical and deterministic accuracies were respectively 80%, 60%, 40%, or 20% of the accuracies achieved with a genetic correlation of 1. This indicates that the deterministic formula can be used to estimate genetic correlations between populations (but does not provide information about the mechanism underlying this correlation); for example when the empirical accuracy is only 60% of the accuracy estimated assuming a genetic correlation of 1, the actual genetic correlation between populations is expected to be 0.6. Using this deterministic formula to estimate the genetic correlation between populations can be especially attractive when only one of the populations has a small number of genotyped individuals.

Overestimation of accuracies with the formula using population parameters for the across-population scenarios is probably due to the inability of the SNPs to capture all the genetic variance in the selection candidates [39,40], which is an underlying assumption of this formula. The empirical accuracy was about 80% of the predicted accuracy, both when GWH individuals or MRY individuals were used as selection candidates. This indicates that only 80% of the genetic variance in the selection candidates was captured by the markers in the reference population, due to differences in LD and allele frequencies of QTL between the reference population and the selection candidates. This proportion of the genetic variance in the selection candidates captured by SNPs in the reference population is the maximum accuracy of genomic prediction for those populations based on the used SNP chip [39].

By using an estimation of the genetic variance in the validation population that can be captured by SNPs in the reference population, the formula based on population parameters becomes a useful formula to predict the accuracy of across-population genomic prediction. This formula is very simple to use and can assess expected accuracies before individuals are genotyped. However, an important question remains regarding which values to use for *M*_*e*_ and the genetic correlation. In this study, *M*_*e*_ were estimated based on the variation in genomic relationships between reference and selection individuals around their expectations based on pedigree information. Similarly to the single-population scenario, *M*_*e*_ of the across-population scenarios were estimated based on the relationships across population. Using this approach, an *M*_*e*_ of about 1800 was estimated when GWH individuals were used as selection candidates, and 2400 when MRY individuals were used as selection candidates, both when HF individuals were used as reference population. Since only 10% of the genome was taken into account, this *M*_*e*_ should be multiplied by 10 to get the actual *M*_*e*_ across those populations. In a previous study, an *M*_*e*_ of 11 500 was obtained when reference individuals and selection candidates shared allele frequencies and LD patterns and of 122 000 when reference individuals and selection candidates shared only allele frequencies [18]. Across breeds, allele frequencies are different, but LD patterns may be partly the same, therefore, *M*_*e*_ across breeds was indeed expected to fall within the values of those groups. This suggests that perhaps an *M*_*e*_ of about 20 000 could be used to predict the accuracy of across-population genomic prediction for closely related cattle breeds and an *M*_*e*_ of about 40 000 or more for more distantly related cattle breeds.

The actual genetic correlation between populations, which is needed in the prediction formula, is in practice not known and depends on the traits and populations of interest. However, we showed that this genetic correlation can be estimated quite accurately using a multi-trait model and high-density genotypes. Thus, it may be possible to estimate this genetic correlation in a limited number of animals and to use it to predict the accuracies of genomic selection for different scenarios.

### Empirical accuracies of genomic prediction

The second objective of this study was to investigate the effect of differences in allele substitution effects of QTL between populations, i.e. genetic correlations that differ from 1, on accuracy of across-population genomic prediction. Our results showed that genetic correlations between populations that are smaller than 1 resulted in a reduced accuracy of across-population genomic prediction that is proportional to the genetic correlation.

In this study, it was assumed that SNPs are representative of QTL, i.e. that SNPs and QTL have the same characteristics. Regarding this assumption, we know that for most complex traits, QTL minor allele frequencies are expected to be low [24,41,42]. However, the SNPs on the chip were selected to have an intermediate allele frequency [43], resulting in ascertainment bias of these SNPs. These differences in allele frequencies indicate that, in practice, QTL and SNPs have other characteristics, thereby reducing LD between QTL and SNPs in empirical studies. In our study, QTL were selected from the SNPs on the chip, which did not completely cover the range of expected allele frequencies of the actual QTL. Therefore, LD between QTL and SNPs may be overestimated, which results in higher accuracies of genomic prediction. In a future study, we will investigate the effect of different QTL allele frequencies on the accuracy of multi-population genomic prediction using loci with different allele frequencies and representative of the whole genome.

Another assumption used in this study was that the trait of interest was only influenced by additive effects. Due to the existence of non-additive effects, the average effects of allele substitution depend on the QTL allele frequencies [20], and might therefore be different across populations. In this study, different effects were considered by simulating genetic correlations between populations that differed from 1. In general, empirical studies use additive models for across-population genomic prediction and provide much lower accuracies than those obtained in this study for a genetic correlation of 1, e.g. [9,11]. This suggests that either SNPs do not represent QTL or that non-additive effects are important for the traits of interest in empirical studies, or a combination of both, which is important biological information.

In this study, genetic correlations between populations of 1, 0.8, 0.6, 0.4, and 0.2 were used to simulate phenotypes. Our results showed that genetic correlations between populations can be estimated quite accurately from the data using a multi-trait model. To date, this was done only in a few empirical studies [38,44]. Karoui *et al.* [38] reported estimated genetic correlations between French dairy cattle breeds that ranged from 0 (fertility; Montbéliarde – Normande) to 0.79 (milk; Montbéliarde – Holstein), with only two out of nine estimated genetic correlations above 0.6. These empirical results show that genetic correlation between populations can differ from 1 and depends on the trait of interest.

Results of this study clearly show that genetic correlation between populations is an important parameter for across-population genomic prediction. The true genetic correlation between populations is not influenced by differences in LD between QTL and SNPs. It is worth noting that apart from differences in allele substitution effects, the genetic correlation can also differ from 1 because of different QTL for the same trait. In terms of accuracy, the value of the genetic correlation is important and not the underlying cause of this genetic correlation. In fact, the genetic correlation specifies the maximum accuracy that can be obtained with across-population genomic prediction, provided that the reference population is very large and the number of SNPs is large enough to find a consistent linkage phase across populations.

### Effect of number of QTL

The third objective of this study was to investigate the effect of the number of QTL underlying a trait on accuracy of across-population genomic prediction, which was studied using a GBLUP method. The results showed that changing the number of QTL without changing any other parameter had no effect on the accuracy.

In the case of genomic prediction within one population, different studies have already shown that accuracies of genomic prediction using GBLUP do not depend on number of QTL underlying the trait [16,17]. If variable selection models were used for genomic prediction, higher numbers of QTL resulted in lower accuracies [16,17,45]. One of these studies also showed that variable selection models have an advantage over GBLUP when the number of QTL is below *M*_*e*_ in genomic prediction within one population [16]. In across-population situations, *M*_*e*_ is much larger than within one population [18], which suggests that, in those situations, it will be easier to have a number of QTL smaller than *M*_*e*_ and, thus it is expected that the use of variable selection models will be beneficial.

## Conclusions

The deterministic formula based on selection index theory, that was derived in this study, can accurately estimate the accuracy of across-population genomic prediction by using population-specific allele frequencies to set-up genomic relationship matrices. Another deterministic formula using population parameters overestimates the accuracy of across-population genomic prediction, because the SNPs in the reference population cannot capture all of the genetic variance in the selection candidates. However, this formula may still be useful because of its simplicity, and is expected to be much more accurate when the proportion of genetic variance in the selection candidates is known with reasonable accuracy and included in the formula. Moreover, the results of this study show that differences in allele substitution effects across populations reduce the accuracy of across-population genomic prediction, with a proportion equal to the correlation between allele substitution effects across populations. The number of QTL underlying a trait does not affect the accuracy of across-population genomic prediction when a GBLUP method is used.

## Appendix

### Proving that $Cov\left({\widehat{a}}_{A_i},{a}_{A_i}\right)=Var\left({\widehat{a}}_{A_i}\right)$ is correct for across-population genomic prediction

The covariance between the predicted and true breeding value of individual *i* of population *A* using a reference population of population *B* is:

A1 $$ \begin{array}{l}Cov\left({\widehat{a}}_{A_i},{a}_{A_i}\right)\\ {}=Cov\left({r}_{G_{AB}}{\upsigma}_{a_A}{\upsigma}_{a_B}\mathbf{g}{\hbox{'}}_{A_i,B}{\left[Var\left({\mathbf{y}}_B\right)\right]}^{-1}{\mathbf{y}}_B,{a}_{A_i}\right)\\ {}={r}_{G_{AB}}{\upsigma}_{a_A}{\upsigma}_{a_B}\mathbf{g}{\hbox{'}}_{A_i,B}{\left[Var\left({\mathbf{y}}_B\right)\right]}^{-1}Cov\left({\mathbf{y}}_B,{a}_{A_i}\right)\\ {}={r}_{G_{AB}}{\upsigma}_{a_A}{\upsigma}_{a_B}\mathbf{g}{\hbox{'}}_{A_i,B}{\left[Var\left({\mathbf{y}}_B\right)\right]}^{-1}Cov\left({\mathbf{a}}_B,{a}_{A_i}\right)\\ {}={r}_{G_{AB}}{\upsigma}_{a_A}{\upsigma}_{a_B}\mathbf{g}{\hbox{'}}_{A_i,B}{\left[Var\left({\mathbf{y}}_B\right)\right]}^{-1}{\mathbf{g}}_{A_i,B}{r}_{G_{AB}}{\upsigma}_{a_A}{\upsigma}_{a_B}\\ {}={r}_{G_{AB}}^2{\upsigma}_{a_A}^2{\upsigma}_{a_B}^2\mathbf{g}{\hbox{'}}_{A_i,B}{\left[Var\left({\mathbf{y}}_B\right)\right]}^{-1}{\mathbf{g}}_{A_i,B}\end{array} $$

The variance of the predicted breeding value of individual *i* of population *A* using a reference population of population *B* is:

A2 $$ \begin{array}{l}Var\left({\widehat{a}}_{A_i}\right)=Var\left({r}_{G_{AB}}{\upsigma}_{a_A}{\upsigma}_{a_B}\mathbf{g}{\hbox{'}}_{A_i,B}{\left[Var\left({\mathbf{y}}_B\right)\right]}^{-1}{\mathbf{y}}_B\right)\\ {}={r}_{G_{AB}}{\upsigma}_{a_A}{\upsigma}_{a_B}\mathbf{g}{\hbox{'}}_{A_i,B}{\left[Var\left({\mathbf{y}}_B\right)\right]}^{-1}Var\left({\mathbf{y}}_B\right)\kern0.36em \times \\ {}{\left[Var\left({\mathbf{y}}_B\right)\right]}^{-1}{\mathbf{g}}_{A_i,B}{r}_{G_{AB}}{\upsigma}_{a_A}{\upsigma}_{a_B}\\ {}={r}_{G_{AB}}^2{\upsigma}_{a_A}^2{\upsigma}_{a_B}^2\mathbf{g}{\hbox{'}}_{A_i,B}{\left[Var\left({\mathbf{y}}_B\right)\right]}^{-1}{\mathbf{g}}_{A_i,B}\end{array} $$

Combining equation A1 and A2, results in:

A3 $$ Cov\left({\widehat{a}}_{A_i},{a}_{A_i}\right)=Var\left({\widehat{a}}_{A_i}\right) $$
